# Supplementary material for: The Sarcoptic Mange in Maned Wolf (Chrysocyon brachyurus): Mapping an Emerging Disease in the Largest South American Canid
Source: Pathogens. 2023 Jun 15;12(6):830. doi: 10.3390/pathogens12060830 (PMC10301014; doi:10.3390/pathogens12060830)
Supplement: Supplementary file 1 [file pathogens-12-00830-s001.zip › pathogens-2420172-supplementary.pdf]

Supplementary material of the manuscript entitled "The sarcoptic mange in maned wolf (*Chrysocyon brachyurus*): mapping an emerging disease in the largest South American canid"

Table S1. General data of captured wolves and sarcoptic mange diagnosis.

Individuals	Sex	Weight (kg)	Estimated age	Diagnostic method	Result
Cbr 1	Male	21	5 years-old	Histopathological	Positive
Cbr 2*	Male	32,5	3 years-old	Histopathological	Negative
Cbr 3*	Female	25	4 years-old	Histopathological	Negative
Cbr 4	Male	30	5 years-old	Skin scraping	Positive
Cbr 5*	Female	23	2 years-old	Skin scraping	Negative
Cbr 6	Female	24	5 years-old	Histopathological	Positive
Cbr 7	Male	21	10 months-old	Skin scraping	Positive
Cbr 8*	Male	29	6 years-old	Histopathological	Negative
Cbr 9*	Male	28	4 years-old	Skin scraping	Negative
Cbr 10	Female	28	5 years-old	Histopathological	Positive
Cbr 11	Female	9	7 months-old	Histopathological	Positive
Cbr 12	Female	10	7 months-old	Histopathological	Positive
Cbr 13	Male	15,4	4 years-old	Histopathological	Positive
Cbr 14	Male	28	6 years-old	Skin scraping	Positive
Cbr 48	Female	16,6	4 years-old	Skin scraping	Positive

*Included in G2A (the other were included in G1).
